# Thermo-Sensitive Nanomaterials: Recent Advance in Synthesis and Biomedical Applications

**DOI:** 10.3390/nano8110935

**Published:** 2018-11-13

**Authors:** Paola Sánchez-Moreno, Juan de Vicente, Stefania Nardecchia, Juan A. Marchal, Houria Boulaiz

**Affiliations:** 1 Nanobiointeractions & Nanodiagnostics, Istituto Italiano di Tecnologia, Via Morego, 30, 16163 Genova, Italy; paola.sanchez@iit.it; 2 Department of Applied Physics, Faculty of Sciences, University of Granada, C/Fuentenueva s/n, 18071 Granada, Spain; jvicente@ugr.es (J.d.V.); stefania@ugr.es (S.N.); 3 Excellence Research Unit “Modeling Nature” (MNat), University of Granada, 18016 Granada, Spain; jmarchal@ugr.es; 4 Department of Human Anatomy and Embryology, University of Granada, 18016 Granada, Spain; 5 Biopathology and Medicine Regenerative Institute (IBIMER), University of Granada, 18016 Granada, Spain; 6 Biosanitary Institute of Granada (ibs.GRANADA), SAS-Universidad de Granada, 18016 Granada, Spain

**Keywords:** thermo-sensitive nanomaterials, PNIPAm, thermo-responsive polymers, LCST, thermochromism

## Abstract

Progress in nanotechnology has enabled us to open many new fronts in biomedical research by exploiting the peculiar properties of materials at the nanoscale. The thermal sensitivity of certain materials is a highly valuable property because it can be exploited in many promising applications, such as thermo-sensitive drug or gene delivery systems, thermotherapy, thermal biosensors, imaging, and diagnosis. This review focuses on recent advances in thermo-sensitive nanomaterials of interest in biomedical applications. We provide an overview of the different kinds of thermoresponsive nanomaterials, discussing their potential and the physical mechanisms behind their thermal response. We thoroughly review their applications in biomedicine and finally discuss the current challenges and future perspectives of thermal therapies.

## 1. Introduction

The synthesis of materials at the nanoscale and their use in biomedical applications is now increasingly directed towards providing function for the design of precise engineered systems [1,2]. In the last two decades, the nanomedicine field has been experiencing an unprecedented expansion in the development of novel nanomaterials designed for improving the diagnosis, monitoring, and treatment of several diseases [3,4,5,6]. Among the disparities in the diversified approaches to nanomaterial, environmental stimuli such as temperature, pH, ionic strength, electric, or magnetic field, and so forth have drawn greater awareness to novel ‘smart’ materials fabrication [7,8]. Smart nanomaterials represent one of the most interesting and exciting classes of materials for use as therapeutic platforms in various biomedical fields [9,10]. Specifically, thermo-sensitive nanomaterials stand out for their ability to target pre-selected sites in response to a change in the temperature. This makes them strong candidates to achieve minimally invasive therapeutic interventions [11,12]. The conduction of heat is one of the fundamental energy transport mechanisms in nature. Thermal properties of nanomaterials depend on many factors that are usually considered insignificant in normal materials. In particular, things such as surface properties, interfacial structures, and quantum size or classical size effects all determine thermal transport in nanomaterials in a significant way, leading to carrier scatterings and localization that are otherwise absent or are not obviously evident in normal materials [13,14].

The literature is vast, with exponential growth in the number of articles demonstrating advances in synthesis, characterization, and application of different thermo-sensitive nanoparticles (NPs) [15,16]. Among the wide variety of NPs reported in the literature, magnetic NPs (MNPs), superparamagnetic iron oxide NPs (SPIONs), gold NPs (AuNPs), liposomes, and thermo-sensitive polymers-based NPs such as micelles and nanogels have gained an increased relevance when biomedical applications are envisioned.

Thermo-sensitive nanocarriers are very promising materials for drug delivery systems, analytical and diagnostic devices, and thermal therapy. Nanodelivery vehicles possess distinct advantages over conventional drug delivery methods [17]. Firstly, due to their small size, NPs are able to bypass biological barriers, such as cell membranes and the blood-brain barrier, allowing greater concentrations of therapeutics to be delivered. Secondly, nanocarriers can be functionalized with active targeting agents to allow selective delivery, minimize side effects, and limit the wastage of drugs. Thirdly, when the nanocarriers reach maximum acculturation in the required sites, the carried drugs can initiate triggers for non-invasive delivery of therapeutic agents [18]. Temperature is a typical example of non-invasive “triggers” at the diseased site. With greater understanding of the difference between normal and pathological tissues and new advances in material fabrication, there is a highly promising use of thermo-sensitive nanocarriers for controlled drug delivery in the future [19].

The field of thermal therapy has been growing tenaciously in the last few decades. The application of heat to living tissues has produced a host of well-documented genetic, cellular, and physiological responses that are being researched intensively for medical applications, particularly for diagnosis and treatment of solid cancerous tumors using image guidance. It is thought that tumor tissue is more hypoxic, more acidic, and nutrient-deficient, as compared to normal tissues [20]. These traits may render some cancer cells more sensitive to heat. However, the overexpression of heat shock proteins has also been observed in some cancers (e.g., breast, endometrial, ovarian cancer, etc.) [21]. These proteins may then make cancer cells more resistant to heat-based therapies than expected, so the effects of thermal therapy are not general in all types of cancer [22]. Traditional hyperthermia has been used in combination with chemotherapy and/or radiation therapy for the eradication of a variety of cancer types in several clinical trials [23,24]. In traditional hyperthermia, the region of the body containing the disease is heated to ~40–45 °C, several degrees above physiological temperature (37 °C). Sustained temperatures above 43 °C cause necrosis of cancer cells, which are more heat sensitive than normal tissue. A major problem with the conventional hyperthermia methods is the difficulty of heating a local tumor region (the target) to the desired temperature without significant damage to the surrounding normal tissue. High temperatures above 43 °C can kill a large number of tumor cells, but normal tissues are also severely damaged under these conventional hyperthermia treatments. Therefore, the development of novel hyperthermia systems that are able to discriminate between the target and the surrounding normal tissues is required. Nanotechnology is expected to have great potential to revolutionize current hyperthermia methods [25]. The controlled application of thermal energy to living tissues has proven to be a great challenge, leading to the development of many sophisticated pre-clinical and clinical devices and treatment techniques. 

In this review, we describe the recent advances in nanomaterials that respond to temperature, which is an easy external stimulus to apply. Specifically, we focused on the synthesis, properties and functionalization of thermoresponsive nanomaterials (i.e., NPs) and their main biomedical related applications; drug delivery, thermal therapy, and diagnostic devices. This includes consideration of mechanisms of thermal response. We thoroughly review their applications in current biomedicine challenges and future perspectives.

## 2. Thermosensitive Nanomaterials: Synthesis, Properties and Functionalization

Thermosensitive nanomaterials constitute a particular kind of smart material with special importance in biomedical applications. In most cases, it is the polymeric nature of the nanomaterial that drives its thermoresponsive character, although liposomes have also been reported to be thermosensitive [19]. The most frequently used thermoresponsive polymers are hydrogels. The thermoresponsive character of the polymeric constituent is either based on a critical solution behavior or a shape memory characteristic [26]. In this review, we will focus on the discussion of the first study case.

### 2.1. Physical Mechanism: Critical Solution Behavior

Polymers of interest in this review exhibit a critical solution behavior as a result of temperature dependent polymer-polymer and polymer-solvent interactions. At a critical solution temperature (CST) the polymer solution undergoes separation from one phase to two phases in a transition associated with a volumetric change in the polymer between extended and compacted coil states [27,28]. This change is associated with the temperature dependent solvation of the polymer and the inversion of the free energy of the polymer-solvent system [26]. The fact that many physical properties dramatically change at the CST is behind the vast number of biomedical applications of these materials. For convenience, one can distinguish between negative and positive thermosensitive polymers [27].

Negative thermosensitive polymers have a lower critical solution temperature (LCST). Below the LCST the polymer swells in the solution. Here, the enthalpy term related to the hydrogen bonding between the polymer and the water molecules dominates, and therefore, is responsible for the polymer swelling. Above the LCST, the polymer contracts. Here, the entropy term related to hydrophobic interactions dominates, and therefore, is responsible for the polymer contraction. The LCST is an entropically driven effect. A model example of a polymer with LCST is a polyalkylacrylamide, specifically Poly(*N*-isopropyl acrylamide) (PNIPAm) in water. Below the LCST, PNIPAm chains are soluble in water because of the existence of hydrogen bonds between the polymer (amide groups) and water molecules. Above the LCST, the hydrogen bonds between water and the PNIPAm fail and water molecules are expelled from the polymer network. This particular polymer has an LCST of 32 °C. However, it can be tuned incorporating hydrophilic or hydrophobic moieties in the polymer structure. An early paper by Feil et al. [29] demonstrated that the LCST of PNIPAm can be controlled by simply varying the monomer composition. By and large, when PNIPAm is copolymerized with hydrophilic monomers (e.g., acrylamide or acrylic acid), the LCST increases [30,31]. However, when PNIPAm is copolymerized with hydrophobic monomers (e.g., *N*-tert-butyl acrylamide), the LCST decreases [32,33]. Many other examples on the change in LCST for a wide range of modified copolymers can be found in Table 1 in the paper by Liu et al. [34]. An increase of the LCST, when copolymerized with acrylamide, up to 45 °C has been reported when 18% of acrylamide is incorporated in the polymer. In contrast, a decrease of the LCST, when copolymerized with *N*-tert-butyl acrylamide, down to 10 °C has been reported when 40% of *N*-tert-butyl acrylamide is incorporated in the polymer [35]. Chen et al. [31] fabricated poly (*N*-isopropylacrylamide-*co*-acrylamide) with different feed ratios by radiation polymerization using Co^60^ γ-rays. In their paper, they describe a larger than 10 °C change when increasing the molar percentage of the copolymer from 3 to 16.

The introduction of amino groups to PNIPAm chain raises the LCST and slows down the phase transition [36].

Within the family of PNIPAm polymers one can find many others such as: poly(*N*,*N*-diethylacrylamide) (LCST ≈ 26–35 °C), poly(*N*-CL)-1-hydroxymethyl) propylmethacrylamide (LCST ≈ 30°C) and poly(dimethylamino ethylmethacrylate) (LCST ≈ 50 °C) [27].

There are other LCST polymers of interest today [37], for instance Poly(*N*-vinylcaprolactam), poly(*N*-vinlycaprolactam) (LCST ≈ 25–35 °C), poly[2-(dimethylamino)ethyl methacrylate] (LCST ≈ 50 °C) and poly(ethylene glycol), which is also called poly(ethylene oxide) (PEO) (LCST ≈ 85 °C). Note that PNIPAm presents a lower biocompatibility and larger hysteresis in its phase transition if compared to other polymers such as oligo(ethylene glycol) methacrylate [38].

Positive thermosensitive polymers have an upper critical solution temperature (UCST). Below the UCST, the polymer contracts in the solution while above the UCST the polymer swells. The UCST is an enthalpically driven effect. Typical examples of polymers with UCST are poly(acrylic acid), polyacrylamide or poly(acrylamide-*co*-butylmethacrylate) [39]. They are most commonly prepared from poly(ethylene oxide)-*b*-poly(propylene oxide)-*b*-poly(ethylene oxide) (Pluronics, Tetronics, Poloxamer).

Undoubtedly, LCST polymers are of far more interest in biomedical applications than their UCST counterparts.

### 2.2. Synthesis of Thermoresponsive Polymeric Particles

Polymeric particles can be prepared starting from monomers, polymers, or macrogels [40]. Most frequently, the synthesis starts from vinyl monomers that can be nonionic, anionic or cationic. Another possibility to synthesize polymeric particles is to start from polymer solutions that are emulsified and later crosslinked, or mixed with oppositely charged polymer solutions to form colloidal polyelectrolyte complexes. Finally, microgels can also be fabricated by mechanically grinding a macrogel.

**Scheme 1:**
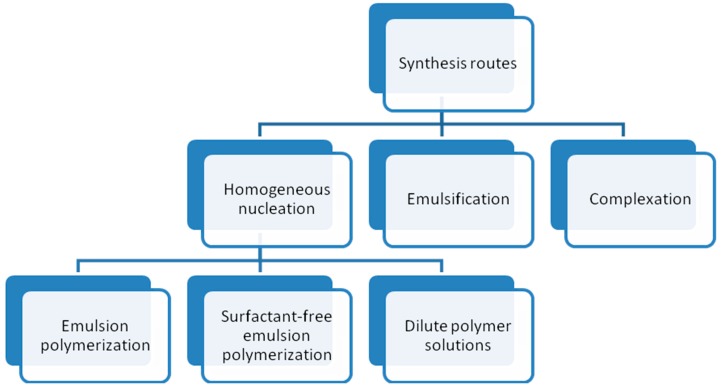
Particle formation mechanism.

In terms of the particle formation mechanism, Pelton and Hoare [40] distinguish three different approaches: Homogeneous nucleation, emulsification, and complexation (Scheme 1).

#### 2.2.1. Homogeneous Nucleation

In this case, the polymer particles are obtained by starting from a homogeneous solution that at least contains one soluble monomer and a cross-linker. For the successful formation of the particles, it is necessary that the resulting polymer is insoluble in the carrier. In this category, we distinguish three different types of polymerization: (i) Emulsion polymerization: the monomer is present as a suspension of large droplets and surfactants play the role of stabilizing the primary particles reducing the size of the grown particle [41]. (ii) Surfactant-free emulsion polymerization (or «precipitation polymerization») is similar to the emulsion polymerization route without the use of surfactants. The approach was inspired by the surfactant-free emulsion polymerization of styrene [42]. (iii) Microgel formation from dilute polymer solutions: the starting point is a polymer solution. For example, this approach has been demonstrated to be successful with PNIPAm [43] and diblock copolymers [44]. Another possibility is to mix polymer solutions having different charges to form complexes.

The use of more than one monomer provides further functionality to the polymer particles allowing, for instance, the fabrication of core-shell structures with monomers having different reactivity. Another possibility is to use emulsion polymerization in a two-step process, provided that nucleation occurs only in the first step and in the second step the first-stage particles are used as seeds for the second-stage.

A typical example of emulsion polymerization is that of McPhee et al. [41]. They prepared aqueous dispersions of PNIPAm by dispersing a copolymerization of NIPAM with methylenebis-acrylamide in the presence of a surfactant (sodium dodecyl sulfate).

A classic example of surfactant-free emulsion polymerization is that of Pelton and Chibante [45] They prepared microgels by free radical polymerization of NIPAM, acrylamide, and *N*,*N*′-methylenebisacrylamide. *N*,*N*′-methylenebisacrylamide was added to give cross-links and acrylamide was used to increase the LCST of the copolymers. The initiator was potassium persulfate.

An example of microgel formation from dilute polymer solutions is that of Kuckling et al. [43]. They used a photo-crosslinking technique to fabricate nanogels with a temperature responsive core and pH responsive arms. They used graft terpolymers synthesized from NIPAAm, poly(2-vinylpyridine) macromonomers and chromophore monomer based on dimethylmaleimide.

#### 2.2.2. Emulsification

Emulsification involves the dispersion and then polymerization of hydrophilic monomers, normally in aqueous solution, in a nonaqueous continuous phase. The emulsifiers employed typically provide the droplets of a steric stabilization mechanism and are different from those of the more conventional oil-in-water emulsion polymerization processes. This is because the electrostatic stabilization mechanism is not effective [46]. Briefly, in the first stage, a pregel solution is emulsified (typically in oil). The pregel can be either a monomer or a polymer solution. Next, the droplets are polymerized and/or crosslinked into the polymeric particle. This kind of polymerization is also typically called «inverse emulsion polymerization» or «miniemulsion polymerization». In some cases, the droplets suffer a homogeneous polymerization while in other cases new particles nucleate within the emulsion droplet. A typical example is that of Landfester et al. [47]. They prepared microgels made of polyacrylic acid using cyclohexane (as the oil phase) and water in the dispersed monomer phase.

Chen et al. [48] studied the kinetic behavior of acrylamide in an emulsification process. The polymerization rate was proportional to the monomer concentration. Increasing the content of the crosslinking agent lead to more monodisperse particles.

The Vincent group [49] used an emulsification approach to fabricate micron-sized microgels on poly-*N*-isopropylacrylamide. In the synthesis, NIPAm, *N*-*N*′-methylenebisacrylamide and the initiator were dissolved in water droplets and later dispersed in heptane (where NIPAm and *N*-*N*′-methylenebisacrylamide are weakly soluble). The stabilizer used was a poly(hydroxy stearic acid)-poly(ethylene oxide)-poly(hydroxy stearic acid) block copolymer.

Another more recent approach is the use of microfluidic devices [50]. Using microfluidic devices the monodispersity of the particles is significantly improved.

#### 2.2.3. Complexation

In this case, two dilute and water-soluble polymers are first mixed to form colloidal polyelectrolyte complexes. In these particular syntheses, one of the polymers must be clearly in excess with respect to the other. An example is described in Feng et al. [51]. They report an investigation into polyelectrolyte complex formation between poly(vinyl amine) and carboxymethyl cellulose. Interestingly, the mean particle size of the complexes was rather insensitive to the mixing ratio. Despite being rather simple, there are two important drawbacks of this method: Resulting microgels are typically polydisperse and in order to be colloidally stable soluble polymer must be in excess. This excess polymer is difficult to separate.

Undoubtedly, the most frequently used method is emulsion polymerization. However, minor modifications allow us to incorporate additional functionalities to the polymeric structure. For instance, seeded polymerization can be used to form a skin layer and introduce shape anisotropy. The pioneering papers describing the synthesis of PNIPAm microgels used precipitation polymerization reactions [45]. More recently, interest has focused on the fabrication of composite multifunctional microgels such as core-shell microgel particles [52,53]. In a conventional drug delivery application, the mechanism behind the de-swelling process plays a key role. In this context, the chemical composition of the shell does have an important influence. Since the shell is typically involved in the initial stages of the de-swelling process, its chemical composition does have an important influence. Gan and Lyon [54] demonstrated that the core-shell architecture strongly affects the kinetics of the phase transition. Hollow microgels can be easily obtained starting with core-shell structures and simply dissolving the core [52].

### 2.3. Hybrid Particles: Incorporation of Magnetic Field and/or Infrared Radiation Sensitivity

Thermoresponsive nanomaterials of interest today are those that respond to other stimuli as well as to temperature. With this in mind, additional stimuli responsive characteristics (e.g., magnetic) must be coupled to the inherent thermal response [19]. Two of particular interest concerns the response to magnetic fields and near infrared radiation. In order for the nanomaterials to exhibit such a response, either (magnetic) iron oxide or gold is respectively incorporated in the form of NPs to the polymer matrix.

The most common routes to incorporate magnetic and infrared functionality in hybrid particles are as follows [26,40]:“(i) Mixing NPs and polymer particles in suspension. The point here is that the NPs must adhere to the polymer and be small enough to penetrate the polymer (see ref [55] for magnetic NPs and [56] for AuNPs). (ii) Polymer first methods. In this case, thermoresponsive polymeric particles are prepared first to serve as a seed in the precipitation of NPs (see Reference [57] for a general description, Reference [58] for magnetic NPs, and Reference [59] for AuNPs) (iii) Nanoparticle first methods. Magnetic iron oxide/AuNPs are fabricated first, followed by the polymerization of thermoresponsive polymers onto the surface of the NPs (see Reference [60] for magnetic NPs and Reference [61] for AuNPs).”

#### 2.3.1. Magnetic Field: Iron Oxides

Magnetic moieties do not necessarily need to be iron oxides. Pure metals such as Fe, Co, and Ni are magnetically superior. However, Fe oxidizes easily in the presence of oxygen and water, Ni is toxic to the body and Co typically introduces coercivity in the magnetic response (i.e., the material remains magnetized in the absence of magnetic fields). As a result, iron oxides (such as magnetite and maghemite) are typically preferred because of their low toxicity and stronger magnetic response compared to other metal oxides.

Either a bottom-up or top-down approach can be used for the synthesis of magnetic NPs [62]. The bottom-up approach is, by far, the most frequently used and among these, the co-precipitation method is the preferred choice to fabricate magnetic NPs. Essentially, ferric and ferrous salts are mixed together in an alkaline media under inert atmosphere. The size, shape and composition of the resulting particles strongly depends on the Fe^2+^/Fe^3+^ ratio, temperature and salts used. As magnetite particles are prone to oxidize, they are typically treated with iron (III) nitrate for their transformation to maghemite, which is now chemically stable.

Other approaches to fabricate magnetic NPs are as follows: Thermal decomposition of organometallic compounds in organic solvents [63], microemulsion approaches [64], and hydrothermal techniques [65].

Magnetic NPs (below 150 nm for magnetite [66]) are superparamagnetic and therefore, tend to aggregate easily unless properly stabilized. For this, the NPs are typically charged, functionalized with citrate ions or covered with surfactants or polymers to provide the repulsion necessary to overcome interactive attraction forces such as van der Waals and magnetostatics.

#### 2.3.2. Infrared Radiation: Gold

A recent review describes the different routes to fabricate thermoresponsive gold-based NPs [67]. They classify the thermoresponsive NPs on the basis of the disposition of AuNPs in the polymer matrix as: hybrid microspheres, hybrid microgel rings, core-shell hybrid microgels, core-shell-hybrid microgels, and yolk-shell hybrid microgels.

Thermoresponsive NPs made of Au are typically fabricated using the following two approaches. One possibility is to use precipitation polymerization at a temperature where the polymer is contracted [68,69]. Another possibility is to use inverse emulsion polymerization techniques in water/oil emulsions [70].

Currently, «polymer first» methods are clearly preferred in the fabrication of hybrid Au microgels. Some examples are as follows: Raula et al. [71] described a synthesis route for the surface functionalization of Au NPs with PNIPAm. The protocol involved the use of reversible-addition-fragmentation chain-transfer (RAFT) polymerization. Wang et al. [72] described the synthesis of PNIPAm + Au composites. The procedure consisted first of the fabrication of PNIPAm gels containing Au-reactive functional groups (thiols) within the polymer side chains. In a second step, the AuNPs are formed in the PNIPAm template by complexation of Au^3+^ ions and thiol groups. More recent approaches are those of Suzuki et al. [73] and Shi et al. [74]. Suzuki et al. [73] prepared cationic polymeric particles using aqueous free radical precipitation polymerization from *N*-isopropylacrylamide and 3-(methacrylamino) propyl-trimethylammonium chloride as monomers. The fabricated microgel particles served as templates to synthesize Au nanoparticles in the cationic sites. Shi et al. [74] synthesized poly(NIPAM-*co*-methacrylic acid) microgels by surfactant-free emulsion polymerization. Then, thiol-functionalized microgels were obtained by the 1-ethyl-3-(3-(dimethylamino)-propyl) carbodiimide hydrochloride (EDAC)-mediated amide bond formation between the carboxyl groups in the microgel and amine groups of 2-aminoethanethiol. Finally, hybrid microgels were obtained by in-situ reduction of an Au precursor.

#### 2.3.3. Functionalization

Hybrid thermoresponsive NPs are interesting for many applications dedicated to microactuators, sensors, and bioseparation. They are also of great interest in biomedicine. In this context, they are extensively used in hyperthermia, drug/gene delivery, and tissue engineering applications [75]. For this aim, first the responsive NPs must be chemically linked to the polymer matrix. This is not a trivial task because the pores within the polymer matrix are of similar size to the particles. Second, thermoresponsive NPs must be functionalized to yield multifunctional nanoparticles with enhanced efficacy, while simultaneously reducing side effects, due to properties such as targeted localization in tumors and active cellular uptake [76]. Typically, carboxyl and amine groups are incorporated in the structure and frequently carbodiimide chemistry is used [40]. These approaches include surface initiated polymerizations, electrojet encapsulation, and ionic coupling of chitosan to surfactant groups attached to magnetite [77]. In active targeting applications, the coupling of a specific ligand on the particles is necessary for the NPs to be recognized by the cells at the disease site. For instance, in cancer applications the surface is functionalized with folic acid and targeted at the tumor cells that over-express folate receptors. In another application, non-viral vectors based on thermoresponsive NPs can be complex, with DNA for effective gene transfection [78]. In general, the NPs must be PEG coated and exhibit a positive surface charge for passive targeting applications. The reason for this is that these NPs are expected to accumulate preferentially in tumors for a long duration because of the enhanced permeability and retention (EPR) effect [19].

### 2.4. Properties

Major characteristics of thermoresponsive NPs are a consequence of their small size [79]. NPs of interest in biomedicine typically have a size between 100 nm and 200 nm [80]. For instance, these materials exhibit a large specific surface area (inversely proportional to the diameter of the particles) hence containing a large number of sites for adsorption and desorption processes. Moreover, due to their small size, these NPs are capable of diffusing within the carrier over long distances. Furthermore, physical changes occur rapidly at the CST because the relaxation time of the volumetric change is proportional to the square of the nanoparticle radius [81].

Monodispersity in particle size is typically desired in applications. Currently, available synthesis routes provide highly monodisperse NPs, with very low polydispersity indexes [79]. The polydispersity index is given by the ratio of weight-average diameter to number-average diameter. Interestingly, a good monodispersity results in sharp changes to the physical characteristics at the CST. There are many different techniques to ascertain the size, shape and inner structure in thermoresponsive NPs; light scattering is a well-known technique to explore the change in volume at the CST. In the case of LCST polymers, the scattering is weak below LCST, while the particles scatter in nearly all incident light above LCST. Light scattering is in some cases complemented by electrophoretic mobility measurements. Diffusive light scattering is very frequently employed to study the temperature sensitivity of thermoresponsive NPs. Small-angle neutron scattering (SANS) and small-angle X-ray scattering (SAXS) are also frequently used to study the phase behavior of microgel particles at different length scales [53]. Atomic force microscopy, also in the solvated stated, and electron microscopy are other widely used techniques to study the morphological characteristics of the NPs. For instance, cryo-TEM has been employed to investigate the morphology and the volume transition of core-shell microgels by Ballauf and Lu [53].

The polymer particle charge is also important. It can be determined using conductometric and potentiometric titration [82]. Through electrophoretic mobility measurements, it is also possible to obtain information on the surface charge density [83]. The electrophoretic mobility is also strongly sensitive to the swelling behavior of the polymer particle. When the swelling is slow the mobility becomes high because the effective surface charge is large.

Another important property of these nanoparticles is their thermoresponsive character. It is well known that cancer cells are more susceptible to heat due to their immature vascular system, in contrast to healthy cells that are capable of removing heat more efficiently [77]. As a consequence, immersion baths and heated blankets were used in the past with this aim [84]. With the advent of thermoresponsive NPs, however, heating can be localized in the target cells maximizing therapeutic potential and avoiding complications coming from side effects [19,77].

A very convenient way to heat these particles and therefore the surrounding tissue is by using either magnetothermal or photothermal therapy, involving magnetic fields or near-infrared radiation, respectively. This is so because biological tissues are transparent to electromagnetic fields and infrared radiation. Traditionally, thermal and external stimuli responses (e.g., magnetic and optical) are independently tuned [26].

Traditionally, thermal and external stimulus responses (e.g., magnetic and optical) are independently tuned [26]. However, nowadays it is possible that the thermal response of the NPs can be controlled by their magnetic/optical properties and vice-versa. This makes it possible to combine therapies in cancer treatment, for instance, involving localized (in space and time) heating (i.e., magnetic/photo hyperthermia) (magnetic hyperthermia [85]; photo hyperthermia [86]), drug/gene delivery (drug delivery [87]; gene delivery [88]), and also tissue engineering [19,37].

## 3. Biomedical Applications of Thermal-Nanomaterials

### 3.1. Analytical and Diagnostic Devices

#### 3.1.1. Thermal-Nanomaterials as Diagnostic Devices

**Figure 1:**
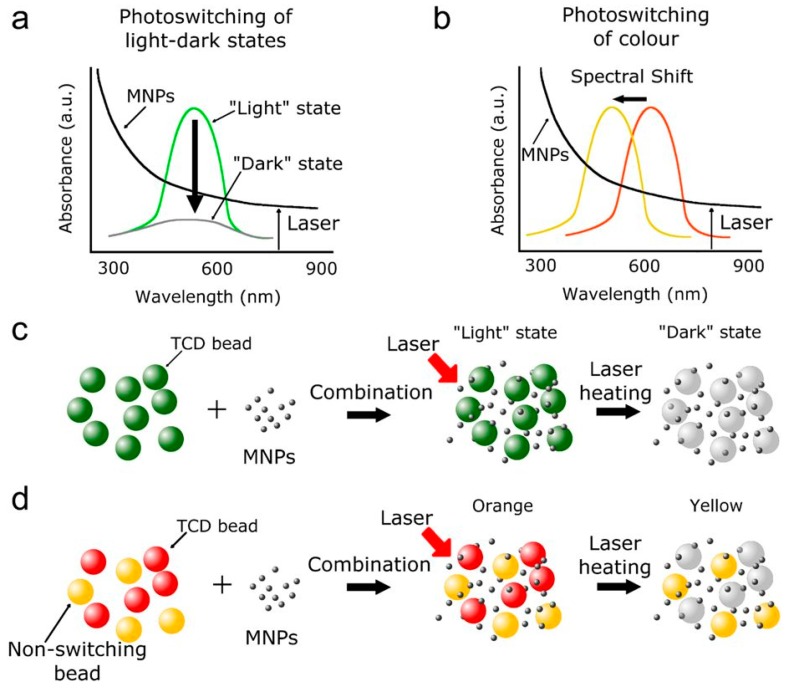
(**a**) Laser-induced temperature-dependent decrease of thermochromic dye (TCD) absorption (“light” to “dark” state transition) during laser heating of embedded magnetic NPs (MNPs). (**b**) Laser-induced temperature-dependent shift in the TCD absorption maximum (blue-shift color). (**c**) Schematic of switching from “light” to “dark” state in monocolor TCD-NP probe. (**d**) Schematic of switching color state in a two color TCD-NP probe. Reproduced with permission from [90]. Copyright Springer Nature Limited, 2016.

Thermo-sensitive materials (metal-based NPs, thermo-sensitive polymers, thermochromic dyes and thermoresponsive nanocomposites) have been investigated for analytical and diagnostic applications, especially in the field of biosensing. Biosensors combine a biorecognition element such as enzymes, nucleic acids, aptamers, etc., with a transducer component that converts the biochemical event into a measurable signal. In the case of thermal biosensors, thermal changes are measured. The interesting phenomenon of thermochromism by which some nanomaterials reversibly switch color as a response to a thermal stimulus is very promising for biosensing. The color-temperature responses may involve the generation or disappearance of color or variations in hue or depth, which can occur very gradually or involve a sudden switch. Some results have been reported in literature, claiming potential in this field, though real applications of this phenomenon in biomedicine are still missing. In line with this, it is worth mentioning the revision work reported by Avella-Oliver and collaborators. This review shows significant bibliographic references that support the potential of thermochromism in biorecognition assays and plots a scenario for new advances [89]. In recent years some new approaches have been reported exploiting chromo-switchable properties of some nanomaterials. Harrington W.N. and co-workers designed a photoswitchable multicolor probe consisting of a thermochromic dye and absorbing magnetic NPs. Light-dark states and spectral shifts in adsorption were reversibly photoswitched by controllable photothermal heating of the NPs (Figure 1). The probes revealed a high contrast sufficient for their visualization in cells and deep tissue, and thus, they could be potentially used in multimodal cellular diagnostics [90].

**Figure 2:**
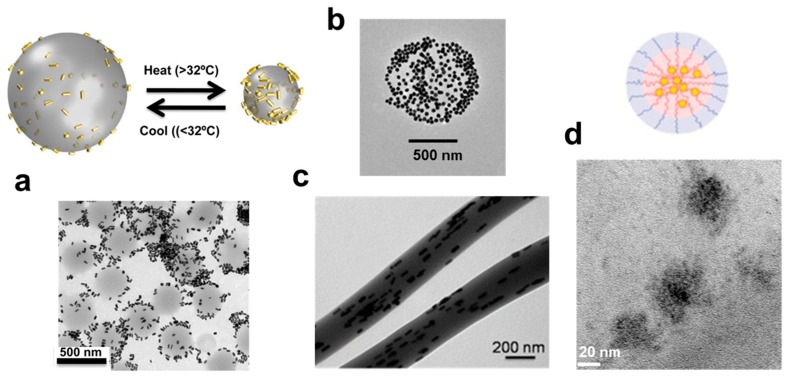
Examples of Au-PNIPAm based thermosensitive nanosystems (**a**) Schematic representation and TEM image of Au nanorods (NRs)–PNIPAm microgel hybrids and the corresponding thermoresponsive behavior (**b**) TEM image of PNIPAm–Au composite (**c**) TEM image of AuNRs/PNIPAm electrospun fibers. (**d**) Schematic representation and TEM image of Au-thermosensitive polymer (PEG-PNIPAm) nanohybrids. Reproduced with permission from [94,95,97,98]. Copyright American Chemical Society, 2015, 2014, 2017, 2018.

Plasmonic NPs combined with materials responsive to temperature have received attention because of their potential applications in drug delivery [91,92], which will be discussed in the next section, and sensing. PNIPAm, the popular temperature-responsive polymer that undergoes a reversible phase transition when heated above its LCST, has been extensively used as a scaffold to control the distance and coupling of plasmonic NPs. Different approaches have been followed: Coating of NPs with PNIPAm, synthesis of the NPs in situ inside PNIPAm matrix or tethering the NPs onto the surface of PNIPAm spheres. This control of the structural parameters of plasmonic nanostructures is critical for their applications, especially in spectroscopy and biosensing [93]. The combination of PNIPAm with gold nanosystems is the most commonly studied (Figure 2).

The greatest significant challenge for these thermoresponsive nanocomposites is to achieve fast responses. In this context, Ding and collaborators recently showed the preparation of AuNP@PNIPAm NPs with increased and faster temperature-induced plasmon resonance shifts compared to that previously reported [94,95], resulting in reversible switching of the optical properties of the Au NPs and holding great potential for temperature sensors [96]. Zhang et al. reported the preparation of Au nanorods (Au NR) and PNIPAm composites with fast thermal/optical response and high heating rate by a traditional electrospinning technique presenting potential applications in smart sensors [97]. Hembury and collaborators showed a synthetic pathway that combined gold nanoclusters with thermo-sensitive diblock copolymers of poly(ethylene glycol) (PEG) and PNIPAm to form a new class of gold-polymer, micelle-forming, hybrid NP [98]. The thermo-sensitive polymers enhanced the native fluorescent signal of Au NPs. This nanocarrier, based on the temperature-dependent, self-assembly of gold nanoclusters within a polymeric micelle core, showed great promise toward bioassays, nanosensors, and fluorescent live-imaging applications.

PNIPAm has also been used in combination with silver NPs because of their high affinity [99,100]. A novel reconfigurable nanocomposite has been recently published, holding promising applications in bio-imaging and color changing technologies. It has been fabricated by incorporating plate-like silver nanoprisms into PNIPAm microspheres and it exhibits a wider range of spectral features and color tenability by changing the size and the loading ratio of the nanoprisms [101].

In the field of cancer diagnosis, new tools are needed in order to identify tumor behavior and improve the treatments. Dual-emission hydrogel NPs have gained much attraction due to the potential they display in biological imaging and detection. The development of a strongly and independently pH-and temperature responsive PNIPAm based hydrogel has been recently reported. In this case, instead of NPs, a red emission pH independent and a blue emission pH dependent molecule with similar excitation wavelengths were inserted in the core and the shell of the hydrogel, respectively. Photoluminescence intensities of the hydrogel NPs showed a linear temperature response (the two emissions), and the blue emission molecule a linear pH response, thus exhibiting different emission colors inside cancer (lower pH) or normal cells. This promises applications for detecting or tracing cancer cells [102]. Moreover, proteases play an important role in cancer; therefore diagnostics that provide information on their activity and function are needed. In this context, thermo-sensitive NPs have been used to design a protease activity nanosensor that can be remotely activated by alternating magnetic fields (AFM) at the site of disease [103]. The nanosensor was composed by thermo-sensitive liposomes and co-encapsulated magnetic NPs and functionalized protease substrates and was used to measure tumor protease activity *in vivo*. The authors of the work demonstrated that this diagnostic tool can identify differences in protease profiles across two *in vivo* human colorectal cancer xenograft models.

#### 3.1.2. Thermal-Nanomaterials as Current Imaging Tests Enhancers

Imaging techniques for the *in vivo* non-invasive diagnosis such as positron emission tomography (PET), single photon emission computed tomography (SPECT), magnetic resonance imaging (MRI), and near infrared fluorescence (NIRF) have made enormous advances in the last decades. Moreover, some types of NPs, used as contrast agents, can significantly improve the resolution of these diagnostic modalities. Improving non-invasive monitoring methods is particularly desirable since current methods of evaluating cell treatments typically involve destructive or invasive techniques, such as tissue biopsies. Some non-invasive methods such as MRI and positron emission tomography (PET), which rely heavily on contrast agents, lack the specificity or resident time to be a viable option for cell tracking. Single photon emission computed tomography (SPECT) allows non-invasive determination of *in vivo* biodistribution of radiotracers at picomolar concentrations. Using specific radiolabelled probes to obtain functional information with high sensitivity about molecular processes is possible. SPECT images have limited spatial resolution and lack anatomical details for reference, making the precise localization of lesions difficult.

New contrast agents for imaging enhancement would facilitate non-invasive monitoring of treatments, allowing simultaneous dynamic imaging of structure and function, and directly provide information on pharmacokinetics and metabolism of drugs. Additionally, they can reduce the overall scan time, avoid multiple anesthesia sessions, and prevent errors associated with coregistration. Moreover, nanomaterials can be used for not only imaging the physical location of cells, but also providing information on the biological state of cells [104]. Many types of NPs are under investigation as potential contrast agents for *in vivo* imaging such as MNPs, for instance, SPIONs [105,106] and AuNPs. In a recent publication, Zhou and collaborators have demonstrated the use of acetylated polyethylenimine (PEI)-entrapped AuNPs (Ac-PE-AuNPs) for precise diagnosis of hepatic carcinoma using negative computed tomography imaging [107]. Magnetically responsive nanosystems present great potential in cancer therapy since MRI can be performed for diagnosis and magnetic guidance under a permanent magnetic field, which can be exploited for tumor targeting. Furthermore, these NPs and some other hybrid nanoassemblies containing thermo-responsive polymers present thermoresponsive behavior, and thus, they are used as multifunctional systems that combine therapeutic and diagnostic capabilities (the so-called theranostics). These nanosystems will be reviewed in Section 3.4.

### 3.2. Thermo-Sensitive Cargo Delivery

In thermal medicine, the procedure of raising the temperature has been used alone to kill cancer cells or as an adjuvant treatment to make surgery or chemotherapy more effective. Raised temperature can provide direct damage, and furthermore, trigger drug or gene release of loaded thermo-sensitive nanocarriers.

Among stimuli-responsive strategies, thermoresponsive drug delivery is one of the most investigated, especially in oncology research. In response to temperature, at least one component of the nanocarriers experiences a change in the properties, triggering the release of the load. Ideally, thermo-sensitive nanomaterials should retain the drug at body temperature (37 °C) and deliver it within the locally heated tumor (40–42 °C) [92].

Thermo-sensitive polymers have been extensively exploited for the synthesis of thermal responsive nanosystems. In the following, we review some examples of different thermo-sensitive nanocarriers investigated in the last five years for the release of different drugs after stimulation with temperature.

#### 3.2.1. Liposomes

Thermo-sensitive liposomes are composed of lipid membranes that undergo phase transitions (from a gel to a liquid phase) in response to heating, at a characteristic phase transition temperature (Tc). Upon heating, the mobility of the lipid head groups, which were ordered and condensed in the gel phase, increases. At the Tc, the hydrocarbon chains switch configuration and the membrane becomes permeable, presenting solid lipid domains and liquid lipid domains. At temperatures higher than Tc, lipids move freely and the bilayers become fully fluidized. During this transition phase, the load of the liposomes is able to leak out.

**Figure 3:**
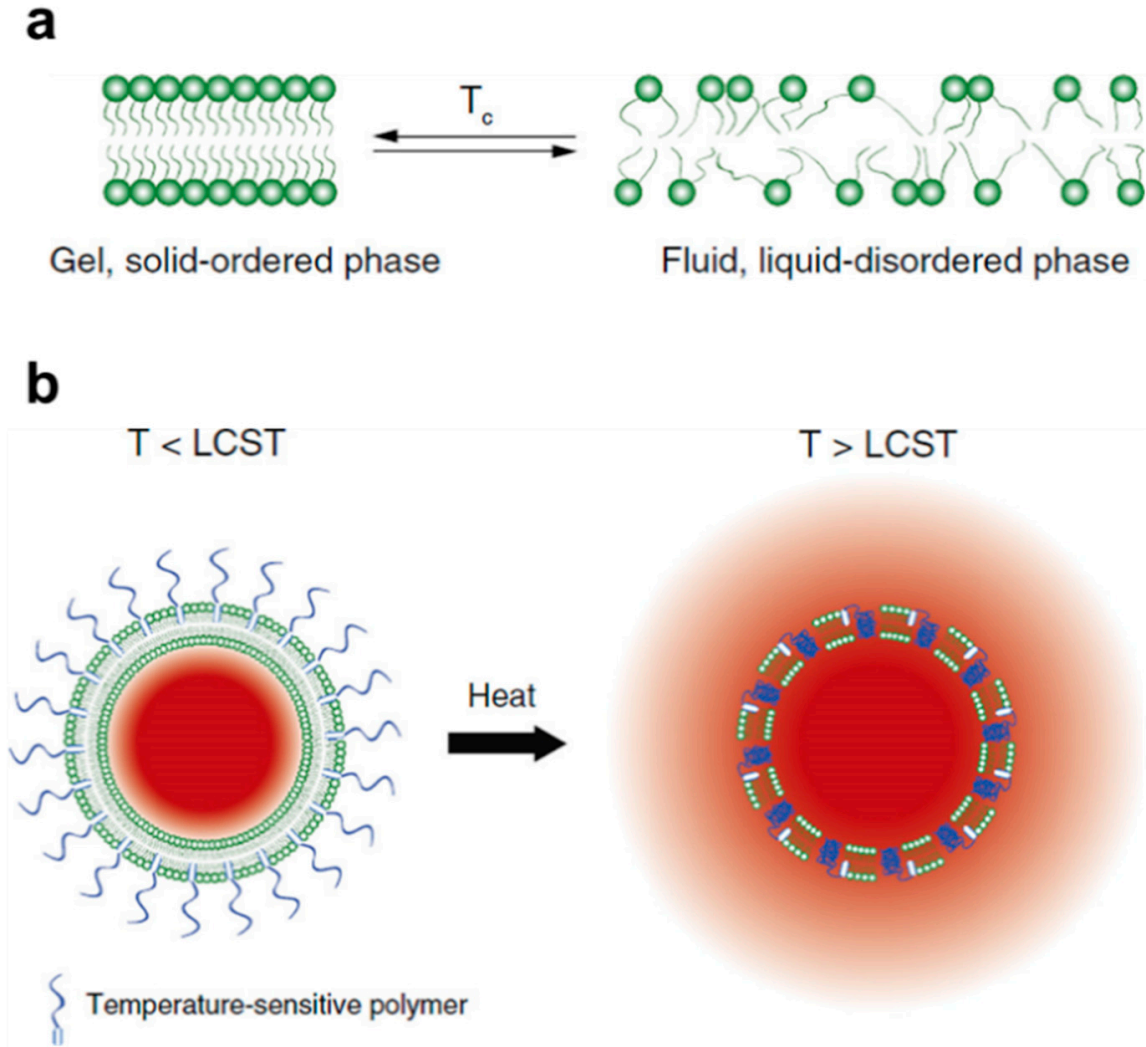
Schematic representation of thermoresponsive liposomes (**a**) Temperature-dependent phase transition of a lipid bilayer. An increase in temperature above Tc is associated with an increase in the lipid bilayer fluidity and an increase in drug release. (**b**) Temperature-responsive polymers disrupt the liposome membrane in response to heating with a consequent increase in drug release. Reproduced with permission from [108]. Copyright Elsevier, 2013.

Non thermo-sensitive liposomes can be sensitized to temperature by functionalizing them with temperature-responsive polymers that disrupt the membrane in response to heating. Such polymers can also enhance the response to temperature of thermo-sensitive liposomes. Temperature-sensitive polymers experience a sharp coil-to-globule transition and phase separation at a LCST or an UCST [108,109] (Figure 3).

To date, thermo-sensitive liposomes for the delivery of doxorubicin (ThermoDox, Celsion) are the most advanced and effective temperature-activated nanocarriers available [110]. Currently, in phase III clinical study, ThermoDox showed a 2.1-year improvement to the overall survival in patients affected with hepatocellular carcinoma. Other liposomal carriers have been recently designed and investigated *in vitro* and *in vivo*. Mild hyperthermia was used to effectively trigger release of cisplatin from thermo-sensitive liposomes in the vasculature of a human model of triple-negative breast cancer (MDA-MB-231 and MDA-MB-436) resulting in a significant tumor growth delay [111]. The liposomes used in this study had a lipid phase transition temperature of 41.5 °C and demonstrated their efficiency in a previous work on mice bearing subcutaneously-implanted ME-180 cervical tumor [112].

Likewise, in another study by Yoon et al. cisplatin was successfully encapsulated in a thermo-sensitive liposomal formulation in order to selectively treat 4T1 murine triple negative breast cancer via photothermal heating. The thermo-sensitive lipid selected was 1,2-Dipalmitoyl-sn-glycero-3-phosphocholine, which has a phase transition of around 41 °C, in order to minimize thermal damage to the body. The loading efficiency was maximized in the formulation called CL16 and exhibited greater therapeutic outcomes, both *in vitro* and *in vivo* [113]. Lv et al. reported a hyaluronic acid-paclitaxel (HA-PTX) prodrug and marimastat (MATT)-loaded thermo-sensitive liposomes for the dual targeting of the tumor microenvironment and breast cancer cells [114]. In this research, they combined the effect of MATT, which avoids metastases by inhibiting enzymes such as collagenases, gelatinases, and matrix metalloproteinases, with the effect of the antitumor drug docetaxel. Thermo-sensitive liposomes were loaded with MATT and assembled with HA-PTX in a multifunctional nanoplatform. The nanocarriers released their payloads after mild hyperthermia treatment at 42 °C and docetaxel entered the cancer cells via CD44 receptor mediation in 4T1 tumor-bearing BALB/c mice.

#### 3.2.2. Micelles

Thermo-responsive polymers have been used to synthetize polymeric micelles that can release the cargo in response to temperature. They are formulated through a self-assembly process using amphiphilic block-copolymers that spontaneously assemble into a core-shell structure in an aqueous environment. The polymers, at either a LCST or an UCST, experience a phase transition that induces collapse of the micelle and the consequent cargo release.

Thermo-sensitive polymers, such as PNIPAm, pluronics (PEG-*b*-PPO-*b*-PEG, triblock copolymers of polypropylene oxide [PPO] middle blocks flanked by polyethylene glycol [PEG] blocks), and poly(hydroxypropyl methacrylamide-lactate) (p(HPMAm-Lac_n_)) are among the most frequently studied. Temperature-responsive micelles have been extensively studied in the past years for their applications as drug delivery systems in cancer therapy. Some formulations have reached clinical trials after showing promising results both *in vitro* and *in vivo*. Fathi and collaborators synthetized chitosan micelles grafted with PNIPAm as temperature-sensitive moiety and oleic acid as hydrophobic monomer. Micelles were targeted with folic acid and loaded with erlotinib, a tyrosine kinase inhibitor [115]. The micelle solution was transparent at 25 °C, which was below the LCST (35 °C). However, with an increased temperature to 37 °C, the solution became opaque and around 90% of the loaded drug molecules were released within 48 h, demonstrating the thermoresponsive behavior of the self-assembled micelles. In a recent study, a biocompatible, degradable and thermo-sensitive amphiphilic polymer PNIPAm-*co*-poly[ethylene glycol] methyl ether acrylate)-block-poly(epsilon-caprolactone) was synthetized and used to produce self-assembled thermo-sensitive micelles loaded with the chromophore cyanine dye IR-780 and heat-shock protein (HSPs, cause of thermotolerance in cancer cells) inhibitors [116]. The controlled drug release during laser irradiation and change of temperature was demonstrated both *in vitro* and *in vivo* in a human colorectal adenocarcinoma cell line.

#### 3.2.3. Core-Shell Nanodevices

Thermoresponsive core-shell nanosystems are a unique class of materials widely studied for drug delivery applications. In general, the thermoresponsive molecule is located on the surface and the core can be constituted by either a hard metallic (gold, magnetic) or a soft (dendrimers, chitosan NPs, silica NPs, nanogels) nanoparticle.

Among the hard metallic NPs, gold nanodevices are the most used due to their photo-inducible heat-generating properties as a consequence of localized surface plasmon resonance (SPR). The heat generated on the surface of the plasmonic NPs causes the thermoresponsive polymer to collapse and the release of the drug. Fathi and co-workers recently designed chitosan copolymer-gold hybrid NPs loaded with erlotinib (ETB), which was released from the nanosystem in a thermo-responsive manner thanks to the temperature-responsiveness of chitosan copolymer composed of (poly(*N*-isopropylacrylamide)-*co*-oleic acid)-g-chitosan ((PNIPAm-*co*-OA)-*g*-CS) that presented an LCST of around 36 °C. The successful cytotoxicity investigation in A549 cells validated their potential as an effective anticancer drug carrier [117]. Au NPs were combined in another study with the copolymer Poly (NIPAAm-*co*-AAm) that was used to create a collapsible thermo-sensitive nanoshell, which exposed targeting ligands (integrin β1) upon NIR irradiation, enabling cell binding. This nanodevice, which exploited the photothermal properties of Au NPs to control NP binding to cell, could be used for targeted photothermal therapy and drug delivery [118]. Magnetic NPs have also been used in combination with different thermoresponsive polymers for drug delivery. Gui and collaborators designed a complex nanodevice constituted of Fe_3_O_4_ NPs/CdTe quantum dots dual-embedded mesoporous silica nanocomposites (MQ-MSN) as cores and P(*N*-isopropylacrylamide)-graft-Chitosan microgels (PNIPAm-*g*-CS) as shells. The carriers, which possessed outstanding magnetism/fluorescence/thermo/pH-sensitivity, were loaded with the anticancer drug adriamycin (ADM) that was released in a temperature dependent manner above the LCST retaining anticancer activity in HepG2 cells [119].

Soft materials like dendrimers have been extensively investigated for the synthesis of thermo-sensitive core-shell NPs. Elastin-mimetic dendrimers were synthetized by conjugating Val-Pro-Gly-Val-Gly repeats, an elastin-like peptide was used as temperature-sensitive biomaterial, to a polyamidoamine (PAMAM) dendrimeric core, though the phase transition temperature presented by the system (48 °C) has to be optimized for biomedical applications [120]. The combination of two dendrimers in the same nanodevice resulted in being more efficient in terms of temperature responsiveness. Oligo (ethylene glycol) (OEG) side chains show attractive thermo-sensitive behavior because their LCST can be tuned from 33 to 64 °C. A thermo-sensitive codendrimer PAMAM-*co*-OEG (PAG) by decorating fourth-generation PAMAM with the second generation OEG dendron was synthetized. This system exhibited high drug (methotrexate) loading capacity and temperature-dependent drug release presenting an LCST of 38.2 °C [121].

#### 3.2.4. Hydrogels

In recent years, thermoresponsive hydrogels are one of the most intensively investigated thermo-sensitive materials for biomedical applications such as drug delivery and tissue engineering and repair. Temperature-sensitive polymer based hydrogels have an LCST above which they undergo transition from a solution to a gel state, forming three-dimensional cross-linked polymeric networks. Injectable biodegradable hydrogels that can form gels in situ have been widely used for biomedical applications, such as cell/drug delivery as well as tissue engineering since they can provide a sustained and controlled delivery to the target site.

**Figure 4:**
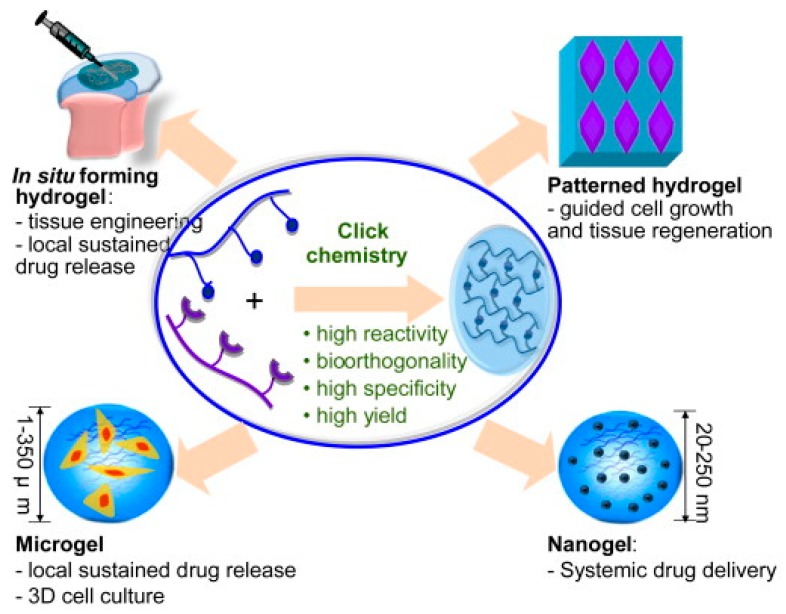
Schematic representation of click hydrogels, microgels, and nanogels synthesis and their potential biomedical applications. Reproduced with permission from [122]. Copyright Elsevier, 2014.

When particles with nanometric sizes are obtained during the hydrogel synthesis, the systems are called nanogels, however, various forms of gels have been studied [122]. Figure 4 shows a schematic representation of the different dimensions and appearances of hydrogels synthetized by click chemistry, a useful approach in forming gels owing to its high reactivity, superb selectivity, mild reaction conditions, and bio-orthogonal feature, though physical and chemical crosslinking methods, mini-emulsion techniques, as well as self-assembly are also important approaches.

Due to the impact of hydrogels on thermo-sensitive biomedical applications, we will review both nanosized hydrogels and hydrogels with other forms and dimensions that are used as thermo-sensitive platforms for the transport of different types of NPs or cells. 

Several natural polymers that demonstrate thermally sensitive properties have been used for the synthesis of hydrogels such as polysaccharides (cellulose, chitosan and xiloglucan) [123,124,125] and proteins (gelatin) [126]. Different polymers such as poly(ethylene glycol)-poly(3-caprolactone)-poly(ethylene glycol) [127], poly *n*-isopropylacrylamide/polyacrylic acid (PNIPAm/PAA) [128], poly(d,l-lactide)-block poly(ethylene glycol)-block-poly(d,l-lactide) (PDLLA-PEG-PDLLA) [129], poly(ethylene oxide)-*b*-poly(propylene oxide)-*b*-poly(ethylene oxide) (PEO-PPO-PEO) [130], and PNIPAm-based hydrogels have also been reported in drug delivery [131,132,133] (see Table 1).

Maiti et al. developed a chemically cross-linked poly-*N*,*N*′-dimethyl aminoethyl methacrylate (PDMAEMA) smart nanogel loaded with both an anticancer drug, doxorubicine, and a radioisotope, ^13^I-labeled albumin, for enhanced chemo-radioisotope therapy. The nanogel in solution form was injected into the tumor where it was transformed into a gel at body temperature. This thermogelling behavior led to the sustained release of the drug and the retention of the radionuclide within the tumor achieving excellent therapeutic *in vivo* results in mice bearing 4T1 tumors [134]. A novel water in water thermo-nanoprecipitation technique for the synthesis of thermoresponsive nanogels composed of dendritic polyglycerol (dPG) and linear thermoresponsive polyglycerol (tPG) as building blocks was recently published. The nanogel was used as a carrier of etanercept (ETR), a protein approved for the treatment of psoriasis and arthritis by subcutaneous injection. Nanogels were topically administered to inflammatory skin equivalents or tape striped human skin, resulting in temperature triggered and efficient ETR delivery and anti-inflammatory effects [135].

Thermal responsive microgels can carry a drug [136,137] or drug-loaded NPs [138,139]. The combination of various drugs in a single microgel can be achieved by encapsulating them directly in the hydrogel [140] or by designing hydrogel composites such as the dual drug loaded thermo-sensitive hydrogel composite recently published by Xu and co-workers [141]. In this study, the anti-tumor effect of the combination of cisplatin-containing thermo-sensitive hydrogel and paclitaxel-loaded polymeric micelles in a single composite (called PDMT) was investigated in an *in vivo* cervical cancer model. Methoxypoly(ethylene glycol)–poly(caprolactone) (MPEG-PCL) was used for the synthesis of the micelles and Poly(ethylene glycol)-poly (epsilon-caprolactone)-poly(ethylene glycol) (PEG-PCL-PEG) for the hydrogel. PDMTs were effective in inhibiting tumor growth and prolonging the survival time of treated mice.

Heat sensitive hydrogels carrying drugs, NPs or cells have been extensively investigated for their application in tissue engineering and regeneration. Recently, vascular endothelial growth factor (VEGF)-loaded poly (lactic-*co*-glycolic acid) (PLGA)-NPs embedded thermo-sensitive gels have been used to promote bladder tissue regeneration in a rabbit model exhibiting favorable performance [142].

Thermo-responsive hydrogels have shown great potential for bone tissue regeneration. Rosuvastatin-loaded chitosan/chondroitin sulfate NPs incorporated into a thermo-sensitive hydrogel provided positive results *in vitro* [143] and methylcellulose hydrogel containing bioactive calcium phosphate NPs showed a higher new bone formation *in vivo* than the pure hydrogel in a rabbit calvaria defect model [144].

There are many [145,146,147,148] *in vitro* and *in vivo* studies demonstrating the effectiveness of hydrogels for central nervous system (CNS) regeneration. Their three-dimensional porous structure is commonly used to load and deliver drugs and growth factors (such as heparin) or they can be injected, successfully inducing bridging of post-traumatic cystic cavities in the spinal cord, as demonstrated by Hong and collaborators. They studied an imidazole-poly (organophosphazenes) (I-5) hydrogel with thermo-sensitive sol-gel transition behavior for the treatment of cystic cavities that develop following injuries to the brain or spinal cord in a clinically relevant rat spinal cord injury model. The dynamic interaction of the hydrogel with the inflammatory cells induced extracellular matrix remodelling to stimulate tissue repair. An improved coordinated locomotion that was accompanied by preservation of myelinated white matter and motor neurons and an increase in axonal reinnervation of the lumbar motor neurons were observed.

Moreover, other thermo-sensitive hydrogels have been investigated as candidates for cardiac regeneration therapy [149,150]. A system based on thermo-sensitive hydrogel and oxygen releasing microspheres was developed for the delivery of oxygen to heart tissue [151]. The system was able to continuously release oxygen for four weeks leading to a significant increase in cardiac function of infarcted rats. Furthermore, different kinds of hydrogels have been developed for skin wound dressings [152,153,154].

**Figure 5:**
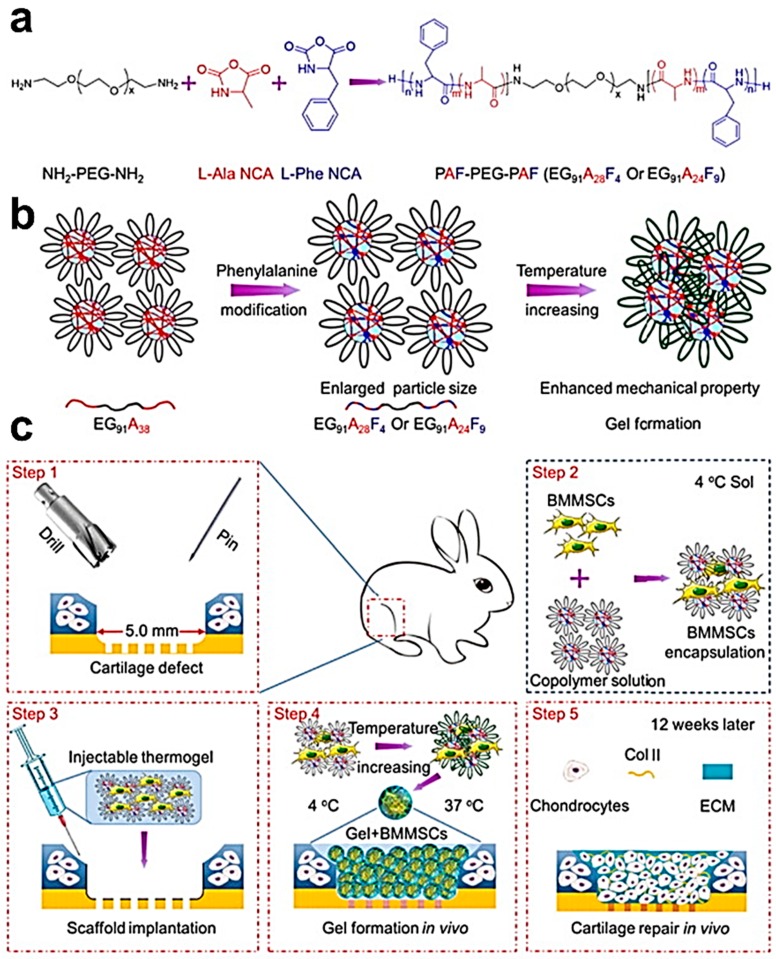
(**a**) Synthetic routes of PAF-PEG-PAF copolymers, (**b**) schematic illustrations of sol-to-gel transition of PAF-PEG-PAF aqueous solutions, and (**c**) procedures regulating BMMSCs chondrogenesis in a rabbit osteochondral cartilage defect model. Reproduced with permission from [156]. Copyright Elsevier, 2018.

Cartilage repair is a great challenge due to the limited capacity for self-healing. Hydrogels with smart sol-gel response for altering environmental temperature have been investigated to promote cartilage regeneration. For instance, a transforming growth factor (TGF)-β1-loaded poly(ε-caprolactone)–poly(ethylene glycol)–poly(ε-caprolactone) (PCEC) hydrogel was fabricated and studied, which demonstrated it to be biodegradable and capable of *in vivo* cartilage repair [155]. Moreover, the regeneration of hyaline-like cartilage with reduced fibrous tissue formation *in vivo* was achieved by Liu and co-workers by increasing the phenylalanine content into a poly(l-alanine-*co*-l-phenylalanine)-block-poly(ethylene glycol)-block-poly(l-alanine-*co*-l-phenylalanine) (PAF-PEG-PAF) thermo-sensitive hydrogel encapsulating bone marrow mesenchymal stem cells (BMMSCs). The increased phenylalanine unit content resulted in an enlarged pore size and enhanced mechanical strength [156]. These features provided better permeability and cell-cell communication, nutrient transportation and cell proliferation, migration, and differentiation that lead to better regeneration of cartilage tissue with reduced fibrous tissue formation (Figure 5).

#### 3.2.5. Polymersomes

Polymer vesicles, also known as polymersomes, are composed of amphiphilic block copolymers that self-assemble into NPs with a hollowed morphology. Due to this vesicular structure, they can encapsulate different loads (such as drugs, peptides and genes) within the hydrophilic hollow or the hydrophobic shell. When temperature-sensitive polymers are used for the preparation of polymersomes, the polymeric drug delivery formulations will be endowed with thermo-sensitive properties and the load will be released after temperature stimulation. Bixner et al. designed magnetic polymersomes by self-assembly of the amphiphilic block copolymer poly(isoprene-*b*-*N*-isopropylacrylamide) (PI-*b*-PNIPAm) with hydrophobic SPIONs. Water soluble dye calcein was encapsulated in the lumen of the vesicles. Magnetic heating of the embedded SPIONs induced a reversible structural change in the polymersome membrane and the hydrophilic compound could be released [157]. In a different study, thermoresponsive diblock copolymers of PEO45-*b*-PtNEAn were prepared and used to synthetize the polymersomes by direct heating of the copolymer solution above the critical aggregation temperature. These aggregates were stable at pH 7.4, but dissociated quickly under mildly acidic conditions resulting in the release of doxorubicin and FITC-labelled lysozyme, which were loaded in the hydrophobic shell and the inner cavity of the polymersomes, respectively [158].

### 3.3. Thermal Therapy

Local hyperthermia is a treatment in which the temperature of the tumor is increased more than other adjacent healthy tissues. In general, the temperature is increased to 40–45 °C, which causes cell damage, and therefore cancer cells become more sensitive to the effects of radiation and anticancer drugs. The methods used for the generation of hyperthermia are microwave [159], ultrasound [160], magnetic [161], near-infrared (NIR) laser, known as photo-thermal therapy (PTT) [162], and radiofrequency [163]. NPs have been investigated as conduits for generating hyperthermia in order to improve uniformity and target specificity of heat in a non-invasive or minimally invasive manner [164]. The most common NPs studied for thermal therapy are MNPs, SPIONs and AuNPs.

Au NPs were investigated to evaluate any synergetic effects of mitoxantrone (MX) and microwave (MW) hyperthermia for the treatment of melanoma. Cell survival was significantly decreased in MX chemotherapy and MW hyperthermia dual treatment compared to the control group [159].

Tissue can be heated through the absorption of ultrasound, which results in acoustic to thermal energy conversion. In order to reduce the ultrasound power or sonication time, and thus, the damage of the surrounding healthy tissues, nanosensitizers can be used since they increase the attenuation and dissipation of the acquired acoustic energy in the form of heat within the tumor area. The efficiency of ultrasound hyperthermia was experimentally and theoretically investigated for anti-cancer treatments by using MNPs in a tissue-mimicking phantom using an ultrasound system [160]. MNPs resulted in being good candidates for sonosensitizing materials in the case of ultrasound-induced hyperthermia.

MNPs are also able to convert energy from an alternating magnetic field to heat, primarily through internal dipole rotation and physical particle rotation, known as Neel and Brownian relaxations, respectively [165,166]. The effects of magnetic hyperthermia induced by two different formulations of biocompatible SPIONs have been studied in breast and pancreatic cancer xenografts in mice, demonstrating an effective tumor growth reduction [167]. A formulation of MNPs loaded with doxorubicin and functionalized with N6L, a molecule that targets a nucleolin-receptor complex overexpressed at the cell surface of tumor cells, was synthetized by Kossatz et al. in order to refine and improve magnetic hyperthermia therapy [168]. The thermally responsive nanosystem selectively targets and successfully eliminates breast cancer cells *in vivo* thanks to the combined effect of chemotherapy and hyperthermia.

As for the other methods for the generation of hyperthermia, the efficiency of radiofrequency (RF)-based therapy can be significantly enhanced by using sensitizers that accumulate in the tumor area and absorb RF radiation power to heat cancer cells. In this case, the nanosensitizers are electrically-conductive NPs that can produce Joule heating through the RF-induced electrical currents over the NP volume [169]. The NPs that provide the strongest absorption of RF radiation are gold [170,171] and carbon nanomaterials [172]. Tamarov and co-workers studied crystalline silicon based nanomaterials (porous silicon-based (PSi) NPs and laser-ablated Si NPs) as sensitizers for RF-induced therapy [163]. Mice were inoculated with lung carcinoma (3LL) cells and treated with the silicon based particles prior to RF exposure. An increase of 20–25% in the lifetime of the mice after the intratumoral injection of both types of NPs was observed. Despite silica NPs having much weaker electrical conductivity than gold NPs, they demonstrated better heating rates.

In photo-thermal therapy (PTT), the photo-sensitizer agents convert the light photon energy absorbed into heat. This method often used light beams with wavelengths in the NIR window (650–900 nm) since maximum tissue penetration is gained. As photothermal agents, a range of metallic and carbon-based NPs with enhanced NIR absorption and high photo stability have been employed [173,174,175,176,177]. However, the heating effect is higher when the beam energy is close to the plasmon frequency of the administered nanosystems. Thus, noble metal NPs such as gold nanostructures with different shapes and gold-based nanoconjugates with different coating molecules that show SPR behavior in the NIR range have been reported [162,178,179].

### 3.4. Theranostics

**Figure 6:**
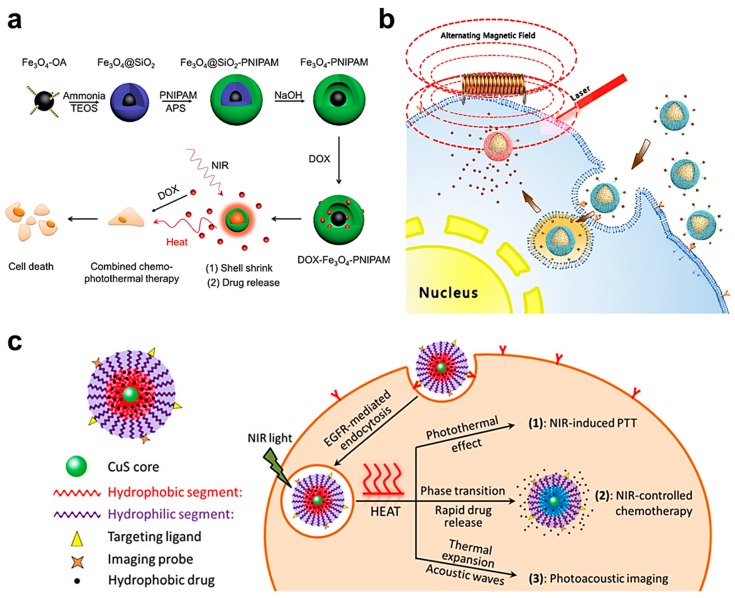
Examples of thermosensitive theranostic nanosystems (**a**) Schematic illustration of the preparation of Fe_3_O_4_-PNIPAm yolk-shell nanocomposites and NIR-trigger drug release. (**b**) Schematic illustration of theranostic functionalities of methotrexate (MTX) modified thermo-sensitive magnetoliposomes (MTX-MagTSLs) loaded with a fluorescent dye Cy5.5 and doxorubicin (Dox). They can achieve dual-imaging effect and provide an appropriate laser irradiation region to release Dox under AMF (**c**) Schematic illustration of a tumor-targeted CuS-based theranostic micelle for combination chemotherapy, photothermal therapy, and photoacoustic imaging. Reproduced with permission from [181,183,187]. Copyright Elsevier, 2017, 2018. Copyright American Chemical Society, 2017.

In nanomaterial-mediated thermal medicine, it is possible to combine imaging that allows for diagnostics and therapy into a single theranostic platform. A broad range of nanomaterials and combinations of different types of imaging (MRI, thermo-acoustic, NIR, thermal, and multimodal imaging) and treatments (drug/gen delivery, heat treatment, chemo and thermo-dual therapy) have been investigated in recent years (Figure 6).

MNPs are among the most studied NPs for the design of thermal theranostics since they represent a tool by being contrast agents for MRI-guided thermoablation of tumors [180,181]. A theranostic nano-platform based on magnetic polydopamine (PDA) coated with hyaluronic acid-methotrexate conjugates and loaded with doxorubicin was synthetized for chemo-photothermal treatment (PTT) of 4T1 tumor-bearing mice. PDA was used as a versatile shell for effective loading of doxorubicin to achieve controlled release and PTT simultaneously. The drug was released from the platform in physiological conditions responding to pH and NIR laser and the active targeting, through magnetic/methotrexate/hyaluronic acid, of the cancer cells increased the cytotoxicity of the tumor cells. NIR fluorescence/MR imaging could be applied to monitor the distribution of the nanosystems [182]. Guo et al. studied a magneto liposome-based theranostic system in which a fluorescent dye Cy5.5, doxorrubicin and MNPs were encapsulated in the bilayer of the liposomes in order to achieve a dual-imaging effect and dual thermo and chemotherapy treatment of cervical cancer. The nanoplatform exhibited temperature sensitivity and responsiveness to AFM and laser simultaneously, which produced hyperthermia triggered drug release and magnetic active targeting. Moreover, the treatment could be monitored in real time by fluorescence and MRI [183]. 

Due to the strong SPR, gold nanomaterials are capable of imaging using techniques like luminescence and computed tomography and at the same time they are potent absorbing agents for converting photons into local heating, as discussed in the previous sections. Thus, they have also been extensively investigated as theranostic platforms for both diagnosis and treatment [184,185]. Shi and collaborators reported an activatable theranostic nanosystem based on activatable aptamer probes (AAPs) and Au@Ag/Au NPs for *in vivo* cancer imaging and guided photothermal therapy in the NIR window. S6 was the aptamer used against A549 cancer cells, showing excellent target recognition ability. The results suggested that the theranostic nanoprobe could be effectively applied for image-guided combined chemotherapy and PTT [186].

Other types of nanomaterials have been investigated as theranostic systems such as micelles [187], liposomes [188,189], nanogels [190], polymer-based NPs [191], quantum dots [192], silica-based NPs [193], black phosphorous-based materials [194], graphene-based materials [195], and so on.

**Figure 7:**
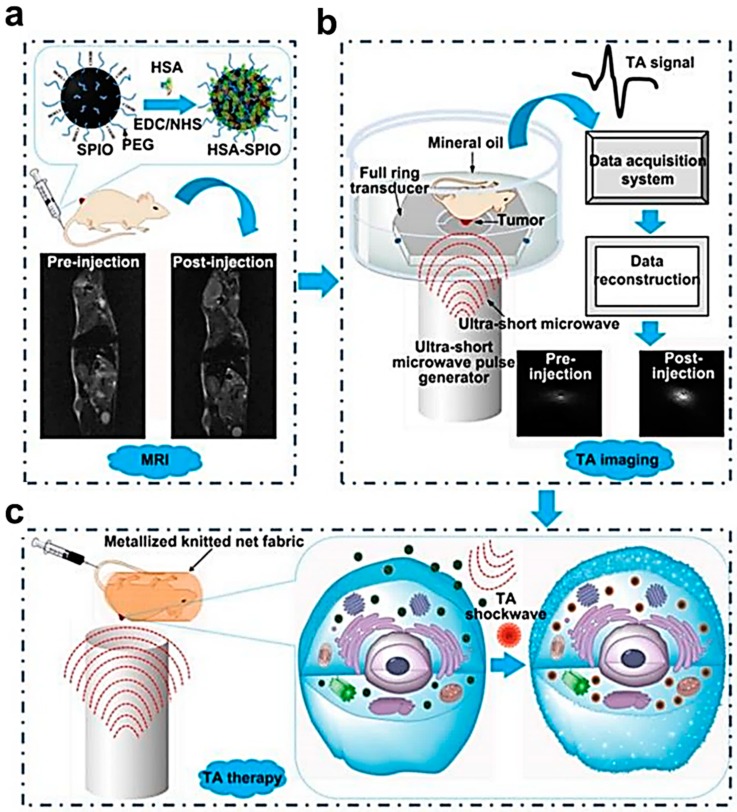
Schematic illustration of (**a**) the process of HSA-SPIO synthesis and intravenous injection into mice for MRI. (**b**) the TA imaging system. (**c**) HSA-SPIO as a TA therapeutic agent for tumor therapy. Reproduced with permission from [196]. Copyright Ivyspring International Publisher, 2017.

A new and promising theranostic approach to tumor imaging and therapy is based on the microwave-pulse induced thermoacoustic (TA) effect, which is based on the conversion of microwave-pulse energy into heat by microwave absorbing agents. The shockwaves produced by the thermal expansion can be acquired by an ultrasound transducer and reconstructed to form TA images. Wen and collaborators have recently studied human serum albumin (HSA) functionalized superparamagnetic iron oxide nanoparticles (HSA-SPIO) as a theranostic nanosystem with potential application for MRI, TA imaging, and treatment of tumors [196]. The authors intravenously injected HSA-SPIO in 4T1 tumor-bearing mice that were localized by MRI. TA imaging was used to indicate HSA-SPIO accumulation in the tumor region that was irradiated with high-energy microwave pulses for treatment. A remarkable inhibition of the tumor growth in HSA-SPIO treated mice was observed (Figure 7). 

## 4. Future Challenges and Perspectives

Temperature sensitive nanomaterials are considered significantly important alongside other stimulus sensitive (pH, light, etc.) nanosystems and they have been widely explored in biomedical applications, but there are still some challenges to face.

One important challenge to overcome, which expands into all nanomaterials, is the clearance and accumulation of NPs in off-target organs such as the liver and spleen. A major reason for the unsuccessful transfer of nanomaterials into the target is that NPs fail to adequately overcome biological barriers and might be sequestrated by cells of the mononuclear phagocytic system, leading to a non-specific distribution that negatively affects the diagnosis and treatment of the tumors. When generating the hyperthermia, it is not possible to target the energy applied to a subset of NPs, and thus, collateral damage in healthy organs while treating cancer cannot be avoided.

In particular, MNPs and hydrogels are among the most commonly thermoresponsive NPs used but present some inconveniences that have to be optimized. MNPs are used as both imaging probes for MRI and as therapeutic agents for magnetic hyperthermia, but the dose of particles required for heating is relatively high and produces saturation of the MRI transverse relaxation time. Thermo-sensitive hydrogels used for cardiac tissue engineering among other applications, however, their ability to mimic native mechanical and electrical properties of the myocardium has to be improved [197]. One of the most widely used polymers for the design of thermal nanomaterials, PNIPAm, presents an LCST close to body temperature and thus is very appealing for biomedicine applications. Despite this advantage, it is non-biodegradable and therefore it has to be copolymerized with other molecules such as PEG or polysaccharides in order to increase its biocompatibility.

On the other side, the use of temperature-sensitive nanocarriers presents enormous advantages. They can provide direct damage of the cells, and hence, the anticancer drug concentration, that in classical chemotherapeutic treatment has to be applied at high doses, thus generating toxicity, can be reduced.

Future directions of thermoresponsive nanomaterials could be their application in the treatment of chronic diseases that need regular doses of the drug to be administered, such as diabetes and the combination of different materials for the multi-modal treatment of cancer. Multiresponsive NPs to different stimuli (light, pH, enzyme, mechanical forces, etc.) could improve the efficiency in both diagnostics and therapeutics. UCST polymers have been under-utilized so far. Future research might be directed to study those polymers that could provide flexibility in designing new thermoresponsive platforms for biomedical applications.

## Figures and Tables

**Table 1:** Thermo-sensitive polymers used for the synthesis of hydrogels.

Thermo-Sensitive Polymers	Characteristics	Some Applications
Poly(ethylene glycol)-poly(3-caprolactone)-poly(ethylene glycol) (PCEC)	High gel strength, slow degradation rate and availability in powder form	Sustained release of bevacizumab in glaucoma filtering surgery. Release of paclitaxel for treatment of cervical cancer
Poly *n*-isopropylacrylamide/polyacrylic acid (PNIPAm/PAA)	Display tunable properties. In slightly acidic conditions, the LCST decreases with increase in PAA content	Variety of molecular switching and drug delivery applications where responses to small pH changes are relevant
Poly(d,l-lactide)-block poly(ethylene glycol)-block-poly(d,l-lactide) (PDLLA-PEG-PDLLA)	Ability to increase the solubility of hydrophobic compounds, extended release of payloads, biodegradability, excellent safety profile	Multidrug (paclitaxel, rapamycin and 17-AAG heat shock protein inhibitor) release for treatment of ovarian cancer. In combination with black phosphorous as a photothermal platform for postsurgical treatment of cancer. Release of growth factors for skin wound healing
Poly(ethylene oxide)-*b*-poly(propylene oxide)-*b*-poly(ethylene oxide) (PEO-PPO-PEO)	Improvement in solubility, stability, release and bioavailability of drugs	Sustained release of drugs for vaginal application. Oral drug delivery. Release of nitric oxide for accelerating wound healing
